# Are Early Somatic Embryos of the Norway Spruce (*Picea abies* (L.) Karst.) Organised?

**DOI:** 10.1371/journal.pone.0144093

**Published:** 2015-12-01

**Authors:** Jiri Petrek, Ondrej Zitka, Vojtech Adam, Karel Bartusek, Naser A. Anjum, Eduarda Pereira, Ladislav Havel, Rene Kizek

**Affiliations:** 1 Department of Chemistry and Biochemistry, Mendel University in Brno, Zemedelska 1, CZ-613 00, Brno, Czech Republic, European Union; 2 Department of Plant Biology, Mendel University in Brno, Zemedelska 1, CZ-613 00, Brno, Czech Republic, European Union; 3 Central European Institute of Technology, Brno University of Technology, Technicka 3058/10, CZ-616 00, Brno, Czech Republic, European Union; 4 Institute of Scientific Instruments, Academy of Sciences of the Czech Republic, Kralovopolska 147, CZ-612 64, Brno, Czech Republic, European Union; 5 CESAM-Centre for Environmental and Marine Studies & Department of Chemistry, University of Aveiro, 3810–193, Aveiro, Portugal, European Union; National Key Laboratory of Crop Genetic Improvement, CHINA

## Abstract

**Background:**

Somatic embryogenesis in conifer species has great potential for the forestry industry. Hence, a number of methods have been developed for their efficient and rapid propagation through somatic embryogenesis. Although information is available regarding the previous process-mediated generation of embryogenic cells to form somatic embryos, there is a dearth of information in the literature on the detailed structure of these clusters.

**Methodology/Principal Findings:**

The main aim of this study was to provide a more detailed structure of the embryogenic tissue clusters obtained through the *in vitro* propagation of the Norway spruce (*Picea abies* (L.) Karst.). We primarily focused on the growth of early somatic embryos (ESEs). The data on ESE growth suggested that there may be clear distinctions between their inner and outer regions. Therefore, we selected ESEs collected on the 56^th^ day after sub-cultivation to dissect the homogeneity of the ESE clusters. Two colourimetric assays (acetocarmine and fluorescein diacetate/propidium iodide staining) and one metabolic assay based on the use of 2,3,5-triphenyltetrazolium chloride uncovered large differences in the metabolic activity inside the cluster. Next, we performed nuclear magnetic resonance measurements. The ESE cluster seemed to be compactly aggregated during the first four weeks of cultivation; thereafter, the difference between the ^1^H nuclei concentration in the inner and outer clusters was more evident. There were clear differences in the visual appearance of embryos from the outer and inner regions. Finally, a cluster was divided into six parts (three each from the inner and the outer regions of the embryo) to determine their growth and viability. The innermost embryos (centripetally towards the cluster centre) could grow after sub-cultivation but exhibited the slowest rate and required the longest time to reach the common growth rate. To confirm our hypothesis on the organisation of the ESE cluster, we investigated the effect of cluster orientation on the cultivation medium and the influence of the change of the cluster’s three-dimensional orientation on its development. Maintaining the same position when transferring ESEs into new cultivation medium seemed to be necessary because changes in the orientation significantly affected ESE growth.

**Conclusions and Significance:**

This work illustrated the possible inner organisation of ESEs. The outer layer of ESEs is formed by individual somatic embryos with high metabolic activity (and with high demands for nutrients, oxygen and water), while an embryonal group is directed outside of the ESE cluster. Somatic embryos with depressed metabolic activity were localised in the inner regions, where these embryonic tissues probably have a very important transport function.

## Introduction

The process of somatic embryogenesis in conifer species (especially the *Picea* and *Pinus* genera) has become a very important tool for the *in vitro* propagation of economically important forest or rare woody species. Due to its potential impact on the forestry industry, there has been rapid development of both a method for their propagation through somatic embryogenesis and a very potent experimental system to investigate the morphological, biochemical, physiological or molecular processes of differentiation and development [1,2,3,4,5,6]. The initiation of embryogenesis has been described in several species of genus *Pinus* (e.g., *P*. *taedea*, *P*. *mariana*, *P*. *roxburghii*, *P*. *bungeana*, *P*. *elliotii*, *P*. *nigra*, *P*. *massoniana*, *P*. *caribaea*, *P*. *silvestris* and *P*. *strobus*), where the process of induction is more difficult in comparison with other conifers, and the genus *Picea* (especially *Picea abies*). *Picea abies* is the first coniferous species (followed by *Larix decidua*) in which zygotic embryos have been used as explants [1,3]. *In vitro* propagation of these species has very important advantages compared to conventional methods because *in vitro* propagation enables the selection of clones with desired attributes and gene transformation [7,8,9]. The process of embryogenesis starts from the induction of embryogenic tissue, usually from the hypocotyls of zygotic embryos. The previous process generated embryogenic cells that eventually formed somatic embryos. Embryogenic cells have common characteristics of meristematic cells with a high division rate (mitotic index) and higher metabolic activity. The embryogenic tissue consists of many somatic embryos that form clusters for use as the source material for the next *in vitro* propagation. However, there is a dearth of information in the literature concerning the detailed structure of these clusters.

Therefore, the main aim of the present work was to determine a more detailed structure of the embryogenic tissue clusters obtained through the *in vitro* propagation of the Norway spruce (*Picea abies* (L.) Karst.). This work also supplied details on the biochemical differences between different areas (inner and outer) of the clusters during two months of cultivation. The outcome of this work will be significant for the subsequent transfer of different cluster regions into new cultivation media and their further development therein, as well as the development of an efficient protocol for the generation of somatic embryos.

## Results and Discussion

### Growth analysis

**Fig 1:**
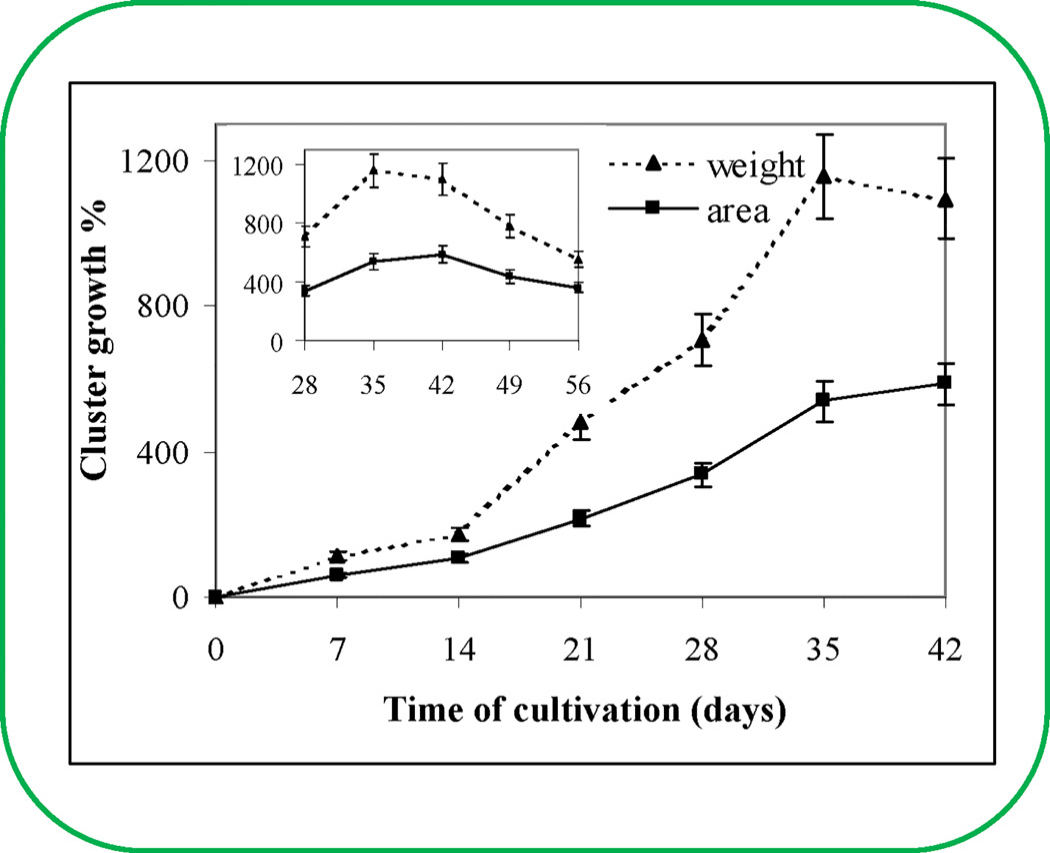
*Picea abies* embryogenic culture. Growth curve of the embryogenic culture over 42 and 56 days (**inset**). The average area of a cluster was 46 mm^2^ and its fresh weight was 110 mg at the beginning of the experiment. The values represent an average of 10 clusters.

The plant growth curve is the basic parameter for a plant physiologist and can be determined using several different methods. The most frequently used methods are based on the counting and/or weighing of cells or tissues. In recent years, image analysis (IA) has been used as a tool to study the growth and/or morphology and also for the study of somatic embryogenesis e.g. *Daucus carota* [10,11,12], *Betula pendula* [13], *Ipomoea batatas* [14], *Saccharum* spp. [15] and *Picea abies* [16]. Therefore, we decided to use this method in this study, too. The ESE clusters cultivated on solid culture medium formed masses with whitish or in some cases slightly yellowish colouring. During the 14-day cultivation, the ESE cluster uniformly expanded to the neighbouring area. The typical structures of individual somatic embryos in the ESEs were observed under a light microscope by very carefully disintegrating the ESEs in phosphate buffer. Each embryo consisted of an embryonal group (very intensely dividing cells), embryonal tube cells and embryonal suspensor cells. Embryonal group cells continually divide, and the tubular cells are formed due to their elongation at the distal end. These cells form the embryonal suspensor, whose cells are released into the medium. The ESE cluster is shaped by the production of new tubular and suspensor cells and their segregation in the direction from the embryonal group [17]. The stress reaction increased after the detachment of 50–100 individual somatic embryos into the cultivation medium, which might account for the growth depression observed for 5–7 days. The growth rate increased from the 7^th^ to 14^th^ day, when very rapid ESE growth was observed. This very rapid growth lasted until the 35^th^–40^th^ day of sub-cultivation (Fig 1). On the 40^th^ day after sub-cultivation, the ESE cluster growth slowly decreased (inset in Fig 1). These growth characteristics were observed using two different methods: computer image analysis and weighing of individual ESE clusters of the 2/32 clone (Fig 1). These growth characteristics were repeatedly validated (n = 3). This rapid growth is often detected in ESEs of various species [4,18].

### Metabolic changes

**Fig 2:**
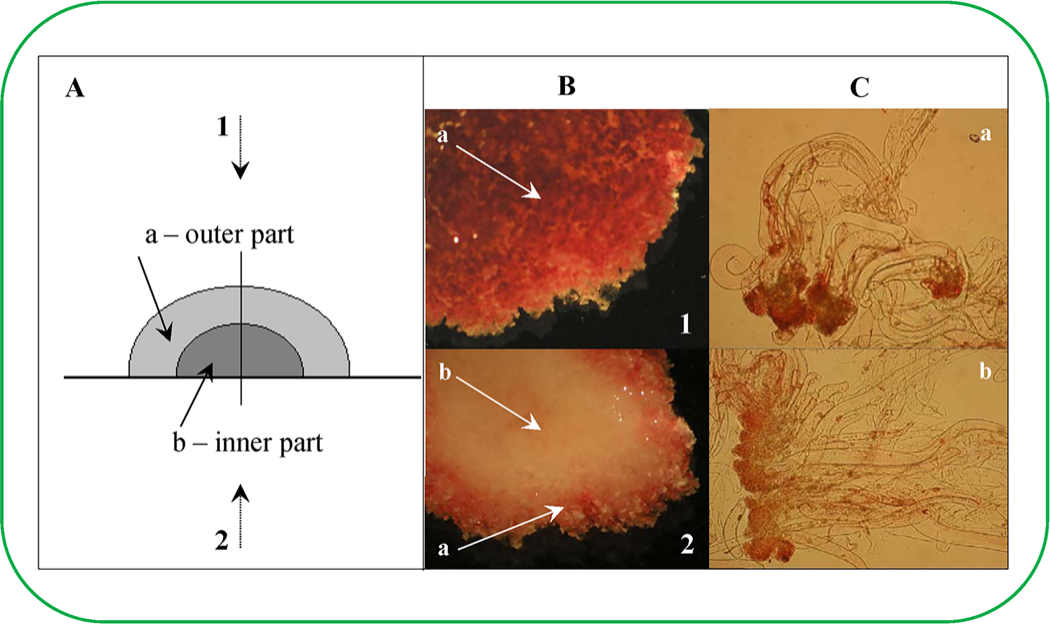
Determination of viability of early *Picea abies* somatic embryos in eight week old clusters (non-vital staining). (**A**) Schematic of the cluster structure of ESEs; (1) view from the top and (2) bottom, (a) inner part of cluster and (b) outer part of cluster. (**B**) Staining with 2,3,5-triphenyltetrazolium chloride (TTC). (**C**) Staining with acetocarmine; (a) embryos from the inner cluster, (b) embryos from the outer cluster.

**Fig 3:**
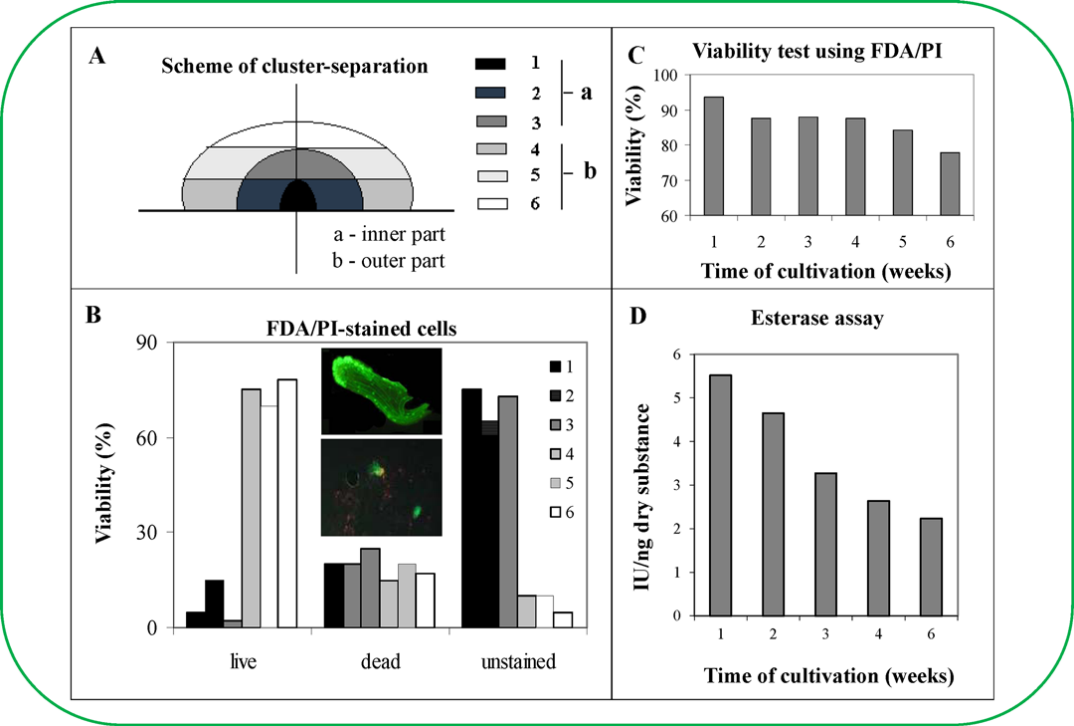
Determination of viability of regions of *Picea abies* embryos (vital staining). (**A**) A six week old cluster of ESEs was divided into six parts for subsequent cultivation. (**B**) Dependence of ESE viability on the cultivation time, which was detected by IA coupled with a fluorescence microscope. Embryos (~1 mg) used for the viability determination were prepared from six regions of the cluster. The cell material was mixed with fluorescein diacetate (FDA) and propidium iodide (PII), and the fluorescence was detected after 5 min by fluorescence microscopy. The percentage of green areas in the compact embryonal group of the embryos (marked by frames) was used to indicate live cells. Insets: upper inset–early somatic embryo (obtained from section 6b) stained with FDA/PI; bottom inset–early somatic embryo (obtained from section 1a) stained with FDA/PI. Average viability changes determined by (**C**) double FDA/PI staining and (**D**) esterase activity determination according to cultivation.

The growth data were interesting and indicated the presence of distinct inner and outer ESE regions (Fig 2A). Significant changes were observed from the 35^th^ day after ESE sub-cultivation. Therefore, ESEs collected on the 56^th^ day after sub-cultivation were selected for experiments to evaluate whether the ESE clusters could be divided into two region to analyse their homogeneity. Individual ESEs were stained with 2,3,5-triphenyltetrazolium chloride (TTC, Fig 2B) and acetocarmine (Fig 2C). The outer cluster was coloured red after TTC staining (Fig 2B), while the inner cluster was unstained. TCC staining indicates changes in the metabolic state of the cell respiratory chain oxidative-reduction reactions. In contrast, acetocarmine stained the embryos of the inner cluster more intensely than the embryos of the outer cluster (Fig 2C), which was based on the measurement of the intensity of the signal. The maximal metabolic activity of the ESE cluster was detected on the surface and in the marginal regions near the ESE cluster base. Individual somatic embryos removed from these regions demonstrated the distinct metabolic activity of the embryonal group. These changes can be associated with changes in recently detected transcription factors [19] or various polyamine levels [20].Furthermore, the ESE cluster regions without metabolic activity (after removing individual somatic embryos) did not demonstrate this activity. Isolated regions of the ESE cluster were subsequently cultivated for 45 days (with sub-cultivations after 14 days) to obtain more information about their viability and metabolic activities. After 45 days of cultivation, the ESE clusters were divided into six parts (Fig 3A). Individual somatic embryos were isolated from each of these parts and subsequently tested for viability using the fluorescent probes fluorescein diacetate and propidium iodide. Nearly 75% of the individual somatic embryos from the inner ESE cluster remained unstained, whereas the number of unstained embryos in the outer ESE cluster was limited to 10%. The number of non-viable cells in each cluster was approximately 15%. These results were confirmed by FDA/PI staining, which demonstrated that individual somatic embryos from the outer ESE cluster demonstrated high metabolic activity and were FDA-positive (Fig 3B). FDA positive clusters were determined based on green fluorescence as it is shown in upper inset photo in Fig 3B. Dead cells were stained by red (bottom inset photo in Fig 3B), whereas unstained cells were identified by superimposing figures with those obtained by standard camera [17]. Average viability changes determined by the above-mentioned double FDA/PI staining and esterase activity determination according to cultivation are shown in Fig 3C and Fig 3D, respectively. There was a gradual deceleration of the ESE metabolic activity that was concomitant with the period of cultivation. This effect could have been caused by the gradual growth of FDA/PI unstainable cells. The potential factor responsible for the metabolic changes observed herein is not clear. Nonetheless, polysaccharide compounds could play a significant role in this process [21,22]. Moreover, there have been observed and documented cases, where the decrease in the metabolic activity of the clusters was associated with the external negative influence of metal ions [23]. Karcz and Kurtyka investigated the effect of cadmium(II) ions on growth, proton extrusion and membrane potential in maize coleoptile segments [24]. The cadmium(II) ions induced growth inhibition at almond seedlings [25], green microalga *Scenedesmus armatus* (Chod.) [26] and others [27,28].

### Metabolite transportation

**Fig 4:**
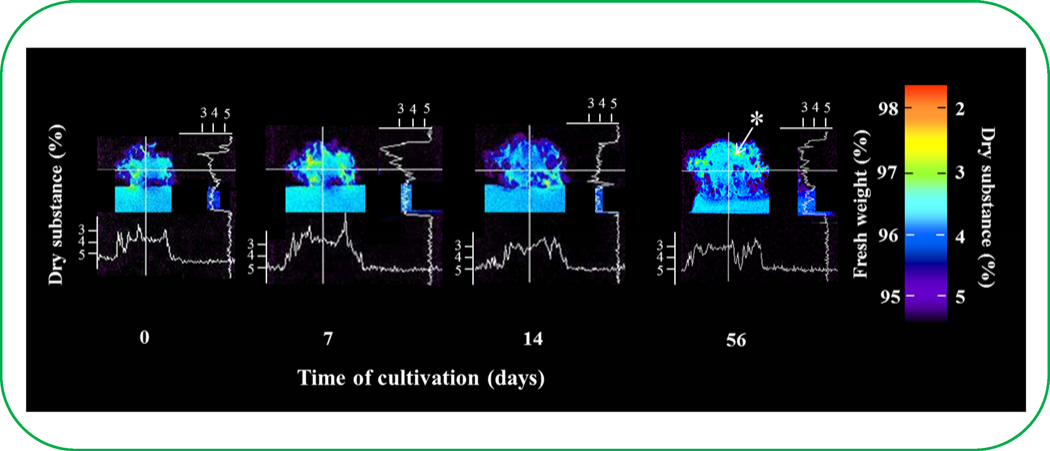
Comparison of the water volume in ESE clusters by NMR. Volume of dry substances in ESE clusters over time. Nuclear magnetic resonance was used to calculate the number of ^1^H nuclei (colour scale). This number corresponds with the volume of water in the ESE clusters (dry substance in clusters). * The formation of a cavity.

This study attempted to describe the differences in the cluster structure and to answer the question of whether the inner ESE cluster structure was connected with the transport of different substances from the cultivation medium into the cluster. In particular, the concentration of ^1^H nuclei was determined by NMR to assess this method of transport. Concentration of ^1^H nuclei determined the concentration of water in ESEs, which was reversely used to find the content of dry matter as it is shown in Fig 4. Scans were taken of the weighted concentration of ^1^H nuclei using MR imaging. A 2.0 mm thick incision was made in the selected sample position, and images were taken of a 30 × 30 mm (256 × 256 pixels) area with a resolution of 0.117 mm/pixel. The samples were cultivated in plastic Petri dishes (50 mm in diameter) for 56 days. Measurements were performed on days 0, 7, 14 and 56 (Fig 4). At the same time, the dry weight was also determined. On the basis of the known dry weight in the cultivation medium, we determined the chromatic scale, dry weight quantity and consequently the water level in each cluster region. The obtained data indicated that there was a higher ^1^H nuclei concentration in the outer ESE cluster compared with the inner cluster. This difference became more pronounced with the increasing cultivation time. These changes are associated with the changes in water content, which could be of interest for further studies because the fact that partial drying of somatic embryos at high relative humidity or desiccation in the presence of supersaturated solutions of salt is used to improve the efficiency of germination. Hazubska-Przybyl et al. found that ESEs of Norway Spruce used in this study react on the heat stress and their behaviour differed from other ESEs [29]. During the first four weeks of cultivation, the ESE cluster seemed to be compactly aggregated; thereafter, the differences between the ^1^H nuclei concentration in the inner and outer clusters were more evident. Additionally, embryos from both the outer and inner clusters appeared to be visibly different. The formation of a cavity was observed in the ESE clusters adjacent to the cultivation medium after more than 40 days of cultivation (Fig 4) and it is label with an asterisk. Although ESEs from the outer cluster were clearly distinguishable and the embryonal group was centrifugal-oriented, the inner parts of the cluster consisted of the mass formed by ESEs and polysaccharide [30,31,32]. The availability of the polysaccharide most likely inhibited the penetration of the fluorescent probes into the ESEs, thereby disabling the determination of ESE viability by double FDA/PI staining [30].

### Growth of embryos obtained from various structures of the cluster

**Fig 5:**
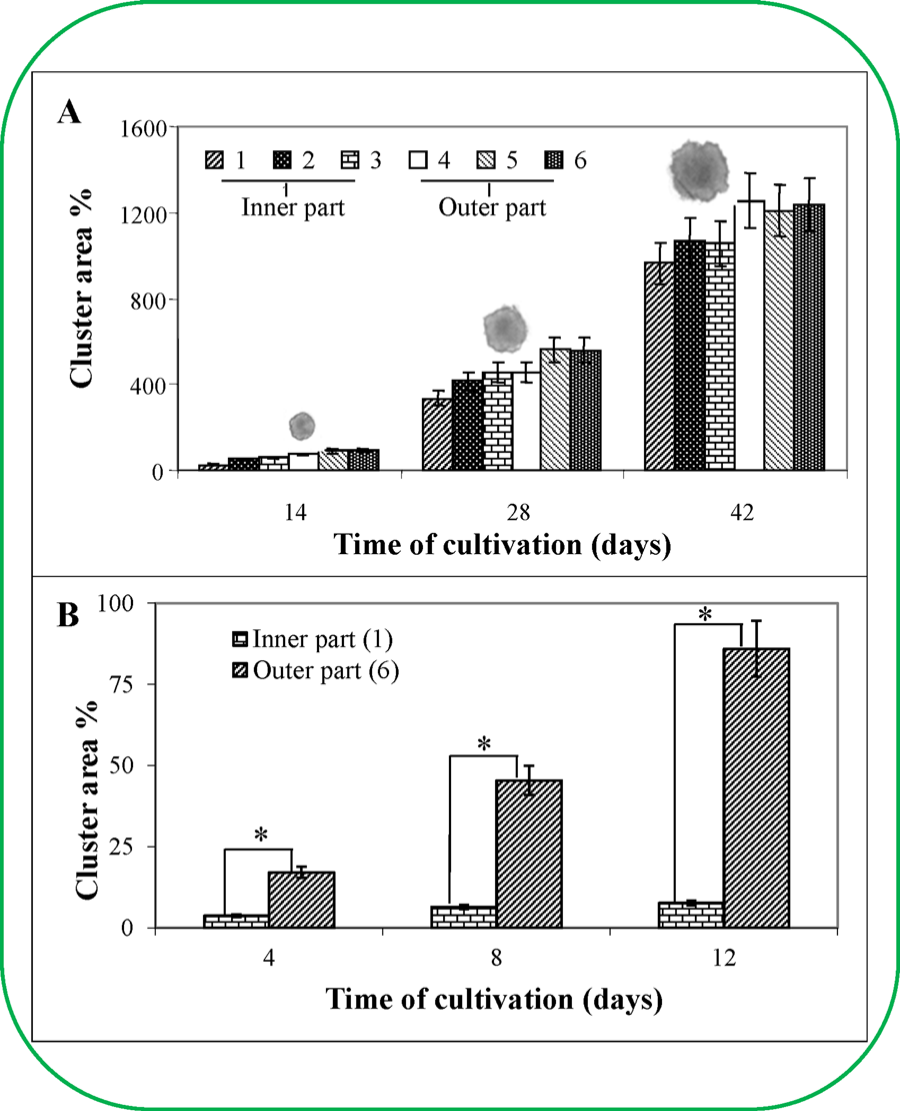
The growth of six regions of the clusters. The growth of six regions of the ESE clusters was characterised by their area in culture medium at 2-week intervals (**A**) over 6 weeks (The average weight of a cluster was 40 mg at the beginning of the experiment. The values represent the average of 10 clusters.) (**B**) or 8 weeks of culture (The average weight of a cluster was 8.6 mg at the beginning of the experiment. The values represent the average of 10 clusters). In inset (A): photos of the representatives of ESEs.

Based on the ESE viability determination, the embryos removed from individual ESE cluster regions cultivated for 45 days (6 parts similar to the viability determination) were placed into new cultivation medium and consequently cultivated for the next 45 days, with sub-cultivations after each two week period. This experiment aimed to determine the cluster area from separate regions of a six-week old cluster. The most evident differences between the individual parts of the cluster were noted during the first 14 days of cultivation (Fig 5A). The outer regions were bigger (over 59.4%) than the inner regions. These differences decreased with the next sub-cultivation. Four weeks after the first cultivation, this distinction between the outer and inner clusters was 31.2%, and six weeks after the first cultivation the difference was only 6.5%. These data clearly indicated that there were differences in the growth characteristics between the outer and inner regions of various locations in the primary cluster (Fig 5A). Each region grew at a particular rate. For example, the inner parts where the older embryos predominated had the ability to grow and develop. The time required to reach the primary growth rate depended on the region of the ESE removed from the primary ESE cluster. As mentioned above, the embryos that originated in the outermost region of the primary six-week old clusters grew almost as quickly as the embryos from the two-week old clusters (the common time of sub-cultivation derived from the growth characteristics; please see Fig 5A). Interestingly, there was an increase in all six regions removed from different locations of the primary cluster (three from the outer and three from inner regions of the primary cluster, photos of the representatives of ESEs are as insets in Fig 5A). It was also evident from the obtained data that the innermost embryos (centripetally towards the cluster centre) could grow after sub-cultivation, although they exhibited the slowest rate and required the longest time to reach the common growth rate. The differences between the individual inner and outer regions (according to Fig 3A) were very obvious and observable, especially during the first 14 days of cultivation. The most prominent differences were between the innermost and outermost regions from the primary cluster surface, where the difference in the growth increase was up to 250% after 14 days of the first cultivation, 35% after 28 days and 30.1% after 42 days. There were found differences in some surface structures in seed of *Pinus sylvestris* L. based on long term experiment with the aim on performing the experiments under various conditions [33]. Based on the obtained data, we could conclude that all embryos from the primary cluster were able to grow and develop despite the fact that vital embryonal cells from the inner regions of the primary cluster seemed to be nonvital due to their lack of double fluorescent FDA/PI staining. Vital (living) cells metabolise FDA to acetate and fluorescein, which is the proper fluorogenic substrate; in contrast, PI penetrates through damaged cytoplasmic membranes and then intercalates into the DNA, causing dead cells to fluoresce red [34]. This is very important because the lack of staining of the inner embryonal cells resulted in our inability to determine their viability despite the fact that these unstained cells were subsequently able to grow and develop.

### Primary cluster

Next, we attempted to determine the age of the primary clusters. By measuring the oxidative-reduction reaction by TTC, we ascertained that the inner part of the primary ESE cluster remained unstained 65 days after the first cultivation, indicating that the cells were dead. We performed the analogous experiment with ESE clusters after 45 days of cultivation when the individual clusters were separated into six different parts. We found differences in the increase in the growth area between the inner and outer regions of the primary clusters. The time points chosen for this experiment were 4, 8 and 12 days (Fig 5B). After 12 days of cultivation, the increase in the area of the new clusters removed from the outer primary cluster was increased by 86%, which corresponded to the area increase of the two-week clusters. Conversely, the area increase in the regions removed from the inner primary cluster was 7.7% after 12 days of cultivation. Thus, the embryonal cells originating from the inner regions of the ESE primary cluster grew very slowly after their transfer into new cultivation medium. These results indicate that the ESE characteristics change depending on the age (time of cultivation) of the ESEs. The results were further statistically treated and it was found that differences determined after 4 days long cultivation were significant (p < 0.05, labelled with an asterisk, Fig 5B).

### The effect of the cluster orientation

**Fig 6:**
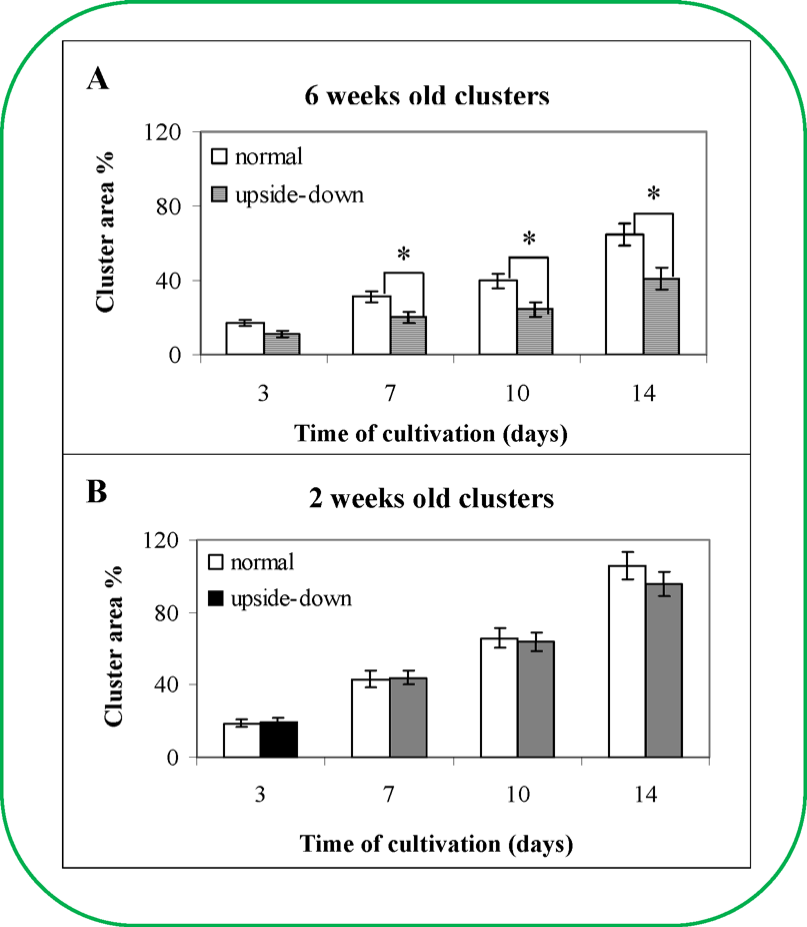
The effect of spatial orientation of the clusters by transposition into new culture medium on the growth of the cluster area after 14 days of cultivation. (**A**) Six week old ESE clusters of clone 2/32 placed in the normal orientation or upside-down in the culture medium after 14 days of cultivation. The average area of a cluster was 190 mm^2^ at the beginning of the experiment. The values represent the average of 5 clusters. (**B**) Two week old ESE clusters of clone 2/32 placed in the normal orientation or upside-down after 14 days of cultivation. The average area of a cluster was 22 mm^2^ at the beginning of the experiment. The values represent the average of 5 clusters.

The water level in different regions of the clusters was determined by employing the NMR technique and was found to be different. Therefore, we hypothesised that each cluster might have various methods for the transport of compounds from the cultivation medium into the cluster. Additionally, we determined the influence of the cluster orientation on the cultivation medium and the change of the three-dimensional orientation of the cluster on its development. We assumed that the change in the ESE cluster orientation would disrupt transport primarily in the older clusters due to the establishment of pathways. For the current experiment, two and six week old clusters were utilised; one half of these clusters were placed into the Petri dish with the same three-dimensional orientation, whereas the second half of the clusters were placed in an inverse orientation (upside down). The areas of the clusters were determined on days 3, 7, 10 and 14 (Fig 6). A difference in the area increase was detectable between the two- and six-week old clusters with the same three-dimensional orientation as the primary cluster (Fig 6A and Fig 6B). When we compared the cluster areas of 6 weeks old clusters, the differences between normal and upside-down were found significant from 7^th^ day of the cultivation (p < 0.05, labelled with an asterisk, Fig 6A). On the other hand, no significant difference was found in 2 weeks old clusters, which could show the differences in structure among two and six weeks old clusters. The area increase after two weeks was approximately 40% higher than the increase observed in the six-week old clusters, which was found significant. This difference was probably due to the diverse ontogenetic stages of the investigated clusters [35]. The regions removed from the older ontogenetic embryos demonstrated slower growth than the regions removed from the two-week-old ESE clusters. However, this experiment was aimed at the investigation of different three-dimensional orientations of clusters in new cultivation medium. The difference between two-week-old clusters with the same orientation and the inverse orientation was approximately 10% after 14 days of cultivation and 25% after 42 days of cultivation (Fig 6). Therefore, it may be necessary to keep ESEs in the same position when transferring them into new cultivation medium.

## Experimental Section

### Chemicals

All reagents were of ACS purity and were purchased from Sigma Aldrich Chemical Corp. (USA) unless otherwise noted. Celulysin was purchased from Calbiochem (USA). All solutions were prepared using deionised water (18.2 MΩ, Iwa 20, Watek, Czech Republic). Culture media were prepared using plant cell culture chemicals purchased from Duchefa Biochemie BV (Haarlem, The Netherlands) unless otherwise indicated. The pH value was measured using the WTW inoLab Level 3 with a level 3 terminal (Weilheim, Germany) controlled by a personal computer program (MultiLab Pilot; Weilheim, Germany). The pH-electrode (SenTix-H, pH 0–14/3 M KCl) was regularly calibrated with a set of WTW buffers (Weilheim, Germany). Deionised water underwent demineralisation by reverse osmosis using the Aqua Osmotic 02 instrument (Aqua Osmotic, Tisnov, Czech Republic) and was subsequently purified using the Millipore RG (Millipore Corp., USA, 18 MΏ) MiliQ water system.

### Plant culture

A culture of early somatic embryos (ESEs) of the Norway spruce (*Picea abies* (L.) Karst.) clone 2/32 was used in the current experiments. The clone was originally established at the Department of Plant Biology of Mendel University in Brno, Czech Republic, according to a procedure described by Durzan et al. [36]. The ESEs were maintained on a semisolid (Gelrite Gellan Gum, Merck, Germany) half-strength LP medium [37] modified by Havel [38,39]. The concentrations of 2,4-dichlorofenoxyacetic acid and N6 benzyladenine were 4.4 and 9 μM, respectively. The pH was adjusted to 5.7/5.8 prior to autoclaving (121°C, 100 kPa, 20 min). The organic part of the medium (except saccharose) was sterilised by filtration through a 0.2 μm polyethylensulfone membrane (Puradisc 25 AS, Whatman, USA). Briefly, ten ESE clusters were cultivated in one plastic Petri dish (100 mm in diameter) containing 30 ml of medium. Sub-cultivation of the stock cultures was performed at 2 week intervals. The stock and experimental cultures were maintained in a cultivation box in the dark at 23±2°C.

### Growth analysis

#### Weighing

The growth of ESE clusters was quantified by weighing on a digital balance (Denver Instrument, APX-153) at different time points. Before weighing, the semisolid medium was carefully cleared off from the clusters using phosphate buffer (pH 7.0). Weighing was performed as fast as possible to avoid desiccation of the clusters.

#### Computer image analysis

A charge-coupled device (CCD) camera was used to observe the growth of the spruce ESE cultures. Images of ESE clusters were recorded at the beginning of the cultivation period and at the indicated time points. The data were converted to digital images by the Grab–IT (version 1.3) program. The area of the ESE clusters in the digital images was calculated by the Image–Pro Plus program (Sony, ver. 1.3). The data were processed in Excel (Microsoft). Details regarding the other procedures used in this study can be found elsewhere [17].

#### Photography of early somatic embryos in bright field

ESEs (~ 0.1 mg) were harvested with a scalpel and transferred to a microscopic slide. The ESEs were spread and superimposed with a cover slip. Subsequently, the sample was placed onto the platform of the microscope (Olympus AX 70, Japan). The images were magnified 40× by the Olympus 4040 digital camera and converted to a digital image in the Grab–IT (version 1.3) program.

### Cell viability assay

#### Double staining

Modified double staining with fluorescein diacetate (FDA) and propidium iodide (PI) was performed to determine the viability of the ESEs [17]. FDA causes green fluorescence in viable cells because the non-fluorescent FDA easily penetrates into viable cells and is hydrolysed to a brightly fluorescent fluorescein (λ_excitation_ = 490 nm and λ_emission_ = 514 nm) that does not readily diffuse through the cytoplasmic membrane. The red fluorescence of PI (λ_excitation_ = 536 nm and λ_emission_ = 620 nm) in cells shows that these cells are dead because this compound cannot pass through the functional cytoplasmic membrane. In our experiments, ESEs (~1.0 mg) were harvested and diluted with water to a final volume of 50 μl. The stock solutions of PI and FDA were added to a final concentration of 20 μg/ml and 1.0 μg/ml, respectively. After 5 min of incubation at room temperature, the percentages of dead (red-stained cells) and viable cells (green-stained cells) were evaluated using an Olympus AX 70 fluorescence microscope with an Olympus cube U MWU coupled with a digital camera. The percent quantification of red (dead cells) and green areas (viable cells) in the compact embryonic group of single embryos was determined from the acquired digital images using an image analysis method (Image–Pro Plus, ver. 1.3, Sony).

#### Metabolic assay

The viability test with 2,3,5-triphenyltetrazolium chloride (TTC) was used to obtain insights into metabolic processes. The TTC metabolic assay exploits enzymatic processes that occur in all living cells and organisms, such as oxidation-reduction reactions during photosynthesis or respiration. In this assay, the clusters of ESEs were incubated in a colourless 1% (*w*/*w*) solution of 2,3,5-triphenyltetrazolium chloride (TTC) for 24 hours. The TTC is taken up by the live cells and reduced to a water-insoluble red formazan by oxidative enzymes. Dead cells remain colourless after incubation with TTC. After 24 hours of incubation at room temperature, the results were photographed using an Olympus (SZH 10) microscope connected to an Olympus (4040 Zoom) digital camera.

#### Esterase assay

ESE cultivation medium was removed by centrifugation (360 × g; 5 min; 20°C; centrifuge: MR 22, Jouan, USA). The cells were washed twice with 50 mM potassium phosphate buffer (pH 8.7). The harvested ESEs (app. 200 mg) were mixed with extraction buffer (250 mM potassium phosphate, pH 8.7) to a final volume of 1.0 ml and homogenised using a Potter_Elvehjem homogeniser (Kavalier, Czech Republic) placed in an ice bath for 10 min. The redox state of the obtained solution was maintained by the addition of 1.0 mM dithiothreitol (DTT). The homogenised samples were sonicated for 1.0 min in an ice bath using a Transsonic T310 sonicator (Czech Republic). The homogenate was centrifuged at 10,000 rpm for 15 min at 4°C (centrifuge MR 22, Jouan, USA). An aliquot (5.0–20 μl) of the supernatant was mixed with potassium phosphate buffer (995–980 μl, 1.0 M, pH 8.75). The reaction was started with the addition of FDA to a final concentration of 5.0μM. The final volume of the reaction mixture was 1.0 ml. An equal volume of the extraction buffer was used as a blank sample. After incubation (15 min, 45°C, dry block, Major Science, Taiwan), an aliquot (between 5.0 to 20 μl) of the reaction mixture was added to 5.0 mM potassium phosphate buffer (pH 8.7, 1980–1995μl). The fluorescence (λ_excitation_ 490 nm and λ_emission_ 514 nm) was read immediately using a spectrofluorometric detector (RF-551, Shimadzu, USA). A stock solution of FDA was prepared in acetone and dried with anhydrous calcium chloride. The amount of acetone did not exceed 1% (*v*/*v*) in the reaction mixture. Esterase activity in international units (IU, one unit liberates one μmol of fluorescein per minute under specified conditions) was recalculated to relative units (100% represents the highest activity measured in an experiment).

#### Acetocarmine staining

The staining methods utilising non-living dyes are based on the penetration of the dyes through disrupted membranes of dead cells and are used to stain cells killed by fixation or directly on living cells. The staining process may also be connected with the tracking of cells killed by the respective staining mixture. This study employed a staining technique based on an aceto-staining agent called acetocarmine solution (solution of carmine in acetic acid). Carmine is a staining agent that penetrates through cell membranes and binds to chromatin. The bound chromatin is stained in bright to dark red colour tones depending on the type of chromatin (i.e., euchromatin and heterochromatin). Compared to other dyes (such as orcein), carmine distinguishes both the chromatin structure and chromosomes during mitosis. In the present experiment, ESEs (~1.0 mg) were harvested and stained without delay with a 1.0% solution of carmine (1.5 g carmine in 100 ml 45% acetic acid; *v*/*v*). Herein, dead and non-living cells were markedly stained red (dark red nucleus, bright pink cytoplasm), whereas living and viable cells exhibited no colour.

### Nuclear magnetic resonance

Tomography based on magnetic resonance was used to study changes in the ^1^H nucleus spin density in ESEs. The experiments were performed on a 4.7 T/120 mm (i.e., 200 MHz for ^1^H nuclei) MRI scanner (Magnex magnet, MR Solution electronics and software) at the Institute of Scientific Instruments, Brno. Actively shielded Magnex gradient coils yielded a maximum gradient field magnitude of 180 mT/m. The samples to be measured were cultivated in a plastic Petri dish (50 mm in diameter). MR images were acquired using a classic Spin Echo pulse sequence with the following parameters: echo time (TE) = 12 ms, repetition time (TR) = 3.8 s, matrix size = 256 × 256 pixels (30 × 30 mm with resolution 0.117 mm per pixel), slice thickness = 2 mm and number of averages of the MR signal was (NA) = 5. The obtained data were processed with the MAREVISI and MATLAB programs.

### Descriptive statistics

Data were processed using MICROSOFT EXCEL® (USA) and STATISTICA.CZ Version 8.0 (Czech Republic) unless noted otherwise. Results are expressed as mean ± standard deviation (S.D.) unless noted otherwise (EXCEL®). Statistical significances of the differences between cluster area and time of cultivation were determined using STATISTICA.CZ. Differences with p < 0.05 were considered significant and were determined by using of one way ANOVA test (particularly Scheffe test), which was applied for means comparison.

## Conclusion

**Fig 7:**
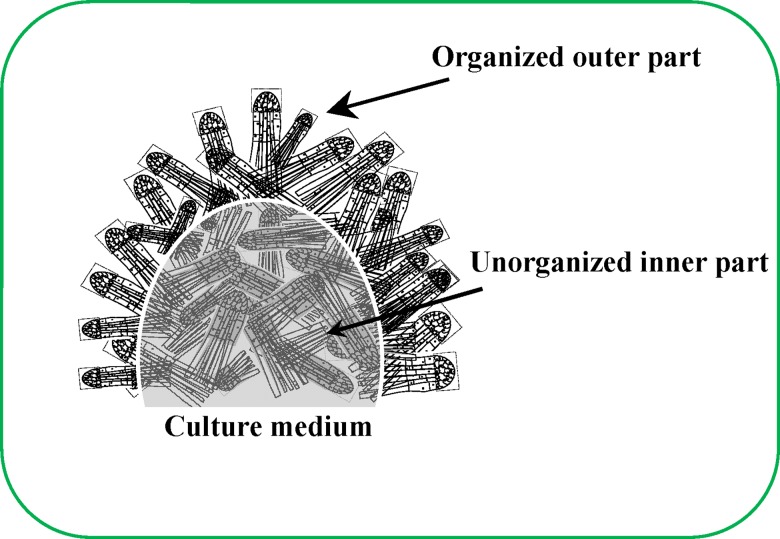
Schematic representation of the structure of the ESE cluster.

The results obtained herein clearly indicate that the transport routes of older clusters are more closely (rigidly) formed and that their interruption can cause growth depression due to the formation of new transport routes. It is also evident that the embryonal cells localised in the inner cluster tend to assume the transport functions for the fully growing ESEs. The possible organisation of this biological structure was suggested on the basis of other experimental data obtained herein. Notably, the outer layer of ESEs can be formed by individual somatic embryos with high metabolic activity (with high demands for nutrients, oxygen and water), whereas an embryonal group is directed outside of the ESE cluster. Somatic embryos with depressed metabolic activity are localised in the inner ESEs, where these embryonic tissues probably have a very important transport function (Fig 7).
